# The effect of psyllium on fasting blood sugar, HbA1c, HOMA IR, and insulin control: a GRADE-assessed systematic review and meta-analysis of randomized controlled trials

**DOI:** 10.1186/s12902-024-01608-2

**Published:** 2024-06-06

**Authors:** Zeinab Gholami, Cain C. T. Clark, Zamzam Paknahad

**Affiliations:** 1 Ph.D Candidate of Nutrition, School of Nutrition and Food Science, Students’ Research Committee , Isfahan University of Medical Sciences, Isfahan, Iran; 2 Department of clinical Nutrition, School of Nutrition and Food Science, Isfahan University of Medical Sciences, Isfahan, Iran; 3 Research Institute for Health and Wellbeing, Coventry University, CV1 5FB Coventry, United Kingdom; 4 Professor of Nutrition, Department of Clinical Nutrition, Faculty of Nutrition and Food Science, Isfahan University of Medical Sciences, Isfahan, Iran

**Keywords:** Psyllium, HbA1c, FBS, HOMA IR, Insulin

## Abstract

There is equivocal evidence that psyllium can prevent or attenuate increases in fasting blood sugar. Therefore, this systematic review and meta-analysis sought to investigate the influence of psyllium on hemoglobin A1C (HbA1c), fasting blood sugar (FBS), insulin, and Homeostatic Model Assessment of Insulin Resistance (HOMA IR). We searched PubMed, ISI Web of Science (WOS), and Scopus for eligible publications, up to 15 July 2022, including randomized controlled trials (RCT) assessing the effect of psyllium on HbA1c, FBS, insulin, and HOMA IR levels in adults. Using a random effects model, we report the weighted mean differences (WMD) with 95% confidence intervals (CI). In this article, 19 RCT studies, consisting of 962 participants, were included. Psyllium significantly decreased FBS, HbA1c, and HOMA IR levels, but not insulin levels, as compared to placebo (FBS: WMD): -6.89; 95% CI: -10.62, -3.16; *p* < .001), HbA1c: (WMD: -0.75; 95% CI: -1.21, -0.29; *p* < .001), HOMA IR: (WMD: -1.17; 95% CI: -2.11, -0.23; *p* < .05), and insulin: (WMD: -2.08; 95% CI: -4.21, -0.035; *p* > .05)). Subgroup analyses illustrated differences in the effects of psyllium on FBS: dosages less than and more than 10 g/d showed significant differences (*p* value < 0.05). However, it was not significant in intervention durations less than 50 days (*p* value > 0.05). For HbA1c: psyllium consumption less than 10 g/d (*p* value > 0.05) was non-significant. For HOMA IR and insulin: no significant changes were noted with psyllium consumption less than vs. more than 10 g/d. In conclusion, we found that psyllium could significantly decrease FBS, HbA1c, and HOMA IR levels, but not insulin levels, as compared to placebo.

**Supplementary Information:**

The online version contains supplementary material available at 10.1186/s12902-024-01608-2.

## Introduction

The global prevalence of diabetes, and in particular type 2 diabetes, is increasing. Ageing and urbanization are factors that are largely attributable for diabetes prevalence in developing countries, however, the resources for treatment are scarce [1] .The word “hyperglycemia” comes from the Greek words hyper (high) and glykys (sweet/sugar), as well as the word haima (blood). Hyperglycemia is defined as blood sugar levels that are higher than 125 mg/dL while fasting and 180 mg/dL two hours after a meal. A patient has pre-diabetes or impaired glucose tolerance if their fasting plasma glucose is between 100 and 125 mg/dL [2].

Empirical evidence suggests that adequate glycemic control is associated with a reduced risk of microvascular (retinopathy, neuropathy and nephropathy) and cardiovascular toll [3, 4].

Dietary fiber has been reported to significantly lower blood sugar levels and increase insulin in people with diabetes [5]. However, it has been asserted that the combination of types of fiber, i.e., dietary fiber (lignin and nondigestible carbohydrates) and functional fiber (nondigestible carbohydrates and isolated) is an important consideration [6].

Psyllium is one of the most beneficial dietary sources of fiber currently available [7], and is a gel-forming mucilage derived from the *Plantago ovata* seed husk [8–10]. The ground skin of psyllium seeds (plantago ovata or psyllium plantago) comprises an admixture of polysaccharides, which includes hexoses, pentoses, and uronic acids, and has been used as a viscose, solvable, gel-forming non-fermented fiber supplement [11]. Psyllium is typically native to India, Iran, and other Middle Eastern countries [12, 13], and the consumption of psyllium seeds has nutritional benefits including, therapeutic treatment of constipation, diarrhea, irritable bowel syndrome, inflammatory bowel disease, ulcerative colitis, colon cancer, diabetes, and hypercholesterolemia. Moreover, psyllium has been posited as a potential therapeutic option for control of diabetes [6–8, 11]. For instance, in one study, psyllium yielded a significant decrease in hemoglobin A1C (HbA1c), as compared to the placebo group, while insulin levels remained unchanged [14]. . Indeed, supplementing a moderate carbohydrate diet with psyllium, even for a short duration, appears to be capable of significantly reducing fasting plasma insulin in those living with diabetes [15]. . By delaying absorption, psyllium has a comparable effect to intestinal α-glucosidase inhibitors in decreasing carbohydrates digestion and absorption, which leads to increased levels of the glucoregulatory factor glucagon-like peptide 1 (GLP-1). In turn, this ensures that vital nutrients arrive to distal regions of the small bowel [6]. A lack of dietary soluble fibers in the diet has been linked to an inexorable rise of coronary heart disease, diabetes mellitus, and colon-related cancer, among other non-communicable diseases [13].

Considering the contradictory findings of studies in the field, we sought to undertake a systematic review and meta-analysis study in order to obtain a more comprehensive result. This study will investigate the effect of psyllium consumption on FBS, HbA1c, HOMA IR, and insulin in adult populations.

## Method

### Search strategy and study selection

The research adhered to the rules and regulations established by the Preferred Reporting Items for Systematic Reviews and Meta-Analyses (PRISMA) guideline [16]. search strategy was done up to 25 march 2022 for PubMed, Scopus and Web of Science (WOS). We found 998 articles in PubMed, 2320 in Scopus, and 1048 in WOS. To assess the effect of psyllium on blood sugar changes HbA1c, Homeostatic Model Assessment of Insulin Resistance (HOMA IR) and insulin, we searched for relevant studies from database inception up to 15 July 2022. across four English language databases (PubMed, Scopus, WOS). The data were carefully retrieved using the following keywords: (“Psyllium” OR “Plant Mucilage” OR “mucilage” OR “lunelax” OR “Metamucil” OR “ispaghul” OR “plantago” OR “isogel” OR “ispaghula” OR “psyllium-husk” OR “Plantago ovata” OR “Psyllium fiber” OR “Plantago psyllium” OR “mucilage polysaccharides”) AND (“Randomized Controlled Trial” OR “Clinical Trial” OR “cluster randomized controlled trials” OR “RCTs” OR “cRCTs” OR “Controlled Clinical Trial” OR “RCT” OR “double-blind randomized controlled trial” OR “Clinical Trials as Topic” OR “clinical trial*” OR “controlled trial*” OR “intervention*” OR “Randomized” OR “Randomized” OR “randomly” OR “single-blind” OR “double-blind” OR “placebo” OR “Pilot study” OR “single-blind randomized controlled trial” OR “Controlled Clinical Trials as Topic” OR “Meta-Analysis” OR “Review” OR “Random Allocation” OR “Single-Blind Method” OR “Double-Blind Method” OR “Cross-Over Studies” OR “Comparative Study” OR “Follow-Up Studies” OR “cross-over” OR “parallel” OR “assignment” OR “trial”) alone or combined together with ‘OR’ and/or ‘AND’. Reference lists of retrieved articles were interrogated for supplementary studies. To ensure accuracy, we carefully restricted our search to only include human subjects. To avoid any potential duplication with endnote software, two independent researchers screened both primary titles and abstracts (Z.Gh. and Z.P.) In addition, we manually searched for additional articles in gray literature from reports, theses, newsletters, site of congress and RCT, and irct.ir. as well as activating the alert system of Scopus and PubMed databases and alert system for the Web of Science database didn’t work but we checked up to 28 September 2023. For articles that we did not have access to, we emailed the corresponding author(s).

### Eligibility criteria

The Population, Intervention, Comparison, Outcomes, and Study (PICOS) criteria were used for this meta-analysis study. Accordingly, population (adults who were over 18 years old), intervention (psyllium), comparison (a control or placebo group), outcome (alteration in FBS, HbA1c, HOMA IR and insulin levels), study design (randomized controlled trials [RCTs]) were included. The following inclusion criteria were assessed: (a) RCTs with either parallel or crossover design (study design); (b) Adults who were ≥ 18 years (Population); (c) evaluated the effect of psyllium on FBS, HbA1c, HOMA IR and insulin changes with a control or placebo group (Intervention, Comparison, and Outcomes). Exclusion criteria were: (a) persons who were less than 18 years old (Population); (b) in vitro, animal, or cell culture studies (Population); (c) articles that were not RCT (study design) (d) studies that were reviews, letters, conferences, and abstracts with defective data, and seminars (study design); (e) defective data (study design); (f) articles without expression standard deviation (SD); (g) articles without a control or placebo group (Comparison); (h) articles whose study duration is less than 2 weeks (intervention duration). (i) Articles that were not in English and (j) Articles that had no baseline mean and SD; (k) studies were conducted in children and adolescents or lactating or pregnant women (Population).

### Data extraction

The endnote software was utilized to record all studies. The data extraction form was completed in both a word processor and spreadsheet by two experienced investigators, (Z.Gh. and Z.P.). All selected papers were thoroughly reviewed by the two researchers, (Z.Gh. and Z.P.). In order to obtain the full-text of the articles that we were not able to access, an email was sent to the corresponding author(s). Following successful full-text review, we extracted the following information: author’s name, the publication year, study location, design of the study (parallel or cross-over), the population of study, mean age of the participants, gender, health status of participants, sample size, psyllium dosage, duration of intervention and the mean ± SD of the FBS, HbA1c, HOMA IR, and insulin levels before and after the intervention. Studies with an additional arm will be reported as separate studies. (Table 1)^(14, 15, 17–32)^. When average and standard deviation were not available in numerical form, the Graph digitizer get data software was utilized to obtain the data from published figures.

### Quality assessment

Two investigators (Z.Gh. and Z.P.) used the Cochrane collaboration’s risk of a bias assessment tool to assess the risk of bias [17]. We assessed seven criteria including for each study including (a) random sequence generation, (b) allocation concealment, (c) blinding of participants and personal, (d) blinding of outcomes assessment, (e) incomplete outcome data reporting, (f) selective outcome reporting, (g) Other potential threats to validity and (h) general risk bias. So, studies were ascribed as low quality (low risk of bias for less than two domains), moderate quality (unclear risk of bias for one or two domains), and high quality (low risk of bias for all seven domains) [17] (Table 2)^(14, 15, 17–32)^. The strength of the evidence presented in the studies was assessed using guidelines established by the Grading of Recommendations Assessment, Development and Evaluation (GRADE) Working Group Using appropriate assessment criteria, we divided the quality of evidence into four levels: very low, low, moderate and high [18].

### Data synthesis and statistical analysis

We sought to assess changes in FBS, HbA1c, HOMA IR, and insulin levels, as calculated from the mean changes and Standard Deviations (SD) using a random-effects model [19]. To accurately measure pooled prevalence estimates with 95% confidence intervals, we utilized a random effects model and Comprehensive Meta-Analysis (CMA) software to assess the degree of heterogeneity between studies. An I^2^ value of more than 50% was used to infer a high level of heterogeneity and may be used as an indication that the random effects model should be applied. To address the sources of heterogeneity, we separately performed meta-regression and subgroup analyses. Meta-regression was used for the dosage of psyllium and duration of studies. In all statistical analyses, the significance level was considered as *P-value* < 0.05, and the meta-analysis was conducted using CMA version 3. If the SD of the mean difference was not available in the published studies, we used this formula: SD change = square root ([SD baseline]^2^ + [SD final]^2^ – [2R × SD baseline × SD final]) [20]. For calculating SD from SE, we used the following formula: SD = SE $\varvec{*}\sqrt{\mathbf{n}}$. When there was no information in the form of average and standard deviation, but it was reported in the form of a graph, the get data Graph digitizer software was used to extract the information. For considering heterogeneity, we used the I square (I^2^) index. Accordingly, (I^2^ < 25%), (I^2^ = 25–50%), (I^2^ = 50–75%), and (I^2^ > 75%) were considered low, moderate, severe, and highly heterogeneous, respectively [21]. We performed pre-defined subgroup analyses based on the baseline FBS, HbA1c, HOMA IR, and insulin levels, psyllium dosage (mg/d), study duration (weeks), persons’ mean age, sample size, health status, sensitivity analysis, and publication bias.

## Results

### Search results

**Fig. 1:**
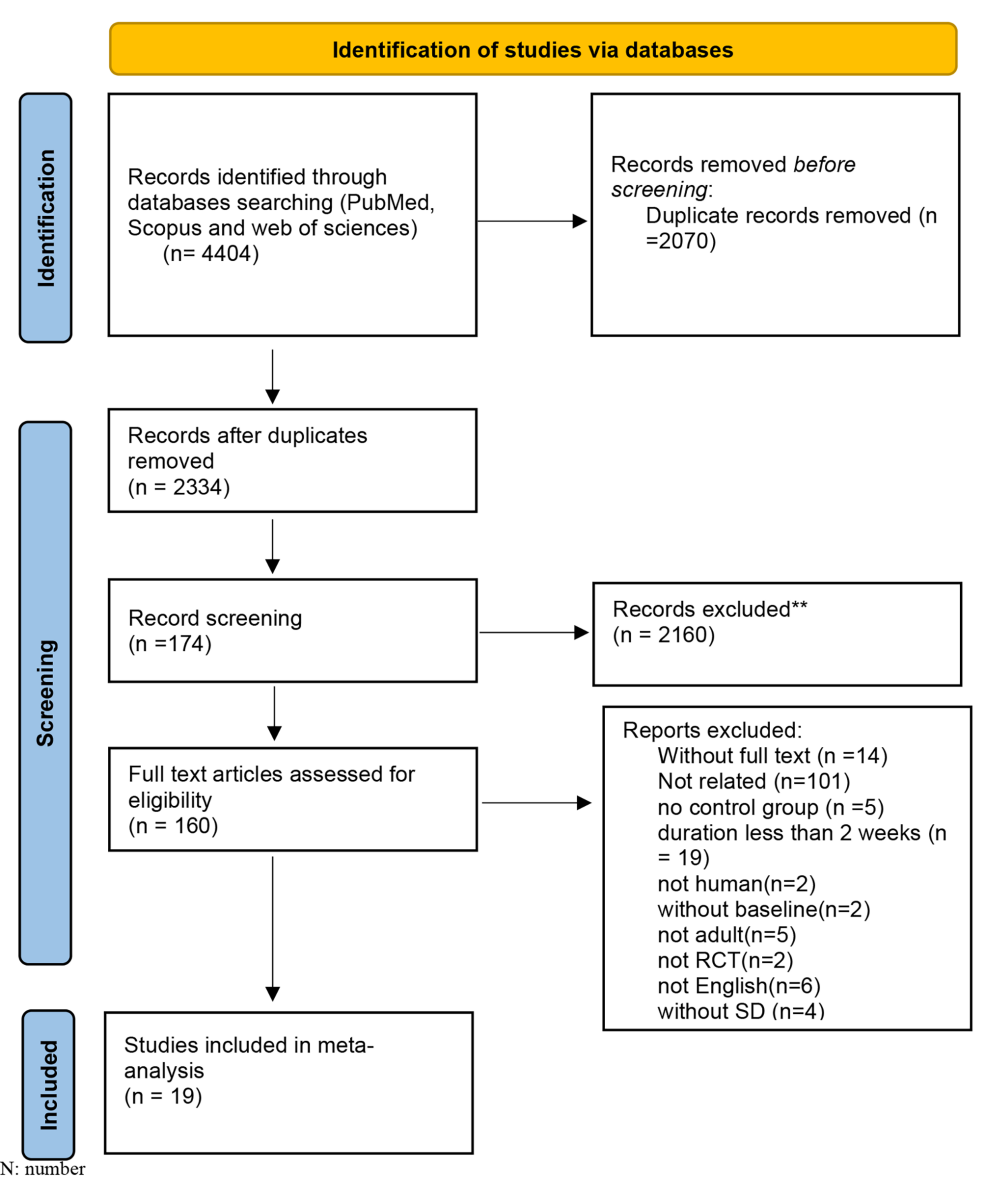
Flow diagram of study selection

This study is registered in PROSPERO, under code CRD42023385375. The flowchart of the procedure of screening and study selection is displayed in Fig. 1. We removed 2334 duplicate articles (2070) and subsequently reviewed the titles and abstracts (2160). Next, 160 full-text articles were screened. We excluded 141 studies, where 42 studies were not related, 5 studies did not have a control group, 2 studies worked on animals, 2 studies had no baseline mean and SD, 5 studies did not work on adults, 6 studies were not written in English, 2 studies were not RCT’s, and 4 studies had no SD. Therefore, 19 RCTs were entered in the final meta-analysis. (Fig. 1)

### Study characteristics

The 19 eligible studies were published from 1985 to 2022 and were 14–182 days in duration. The total number of participants was 962 (481 cases and 467 controls) 962 for FBS, 523 for HbA1c, 575 for insulin, and 591 for HOMA IR. General characteristics of these studies are shown in Table 1^(14, 15, 17–32)^. These studies were conducted in different countries **(**Malaysia, Iran, Palestine, USA, Italy, USA, Spain, Mexico, Pakistan). The mean age of participants ranged between 24 and 77.2 years, with most studies conducted in both genders. The dosages of psyllium utilized in the included studies ranged from 0.002 to 25 g/day.

**Table 1:** Characteristic of included studies in meta-analysis

Author	year	Country	journal	Population	Sex	*N*	In_N	Pl_N	In_Mean Age	Duration (day)	Type of psyllium	Type of Pl.	Dose/day (g / day)
Ong Pui Wen et al [22]	2022	Malaysia.	Journal of Applied Pharmaceutical Science	Healthy male adult	M	29	14	15	26	30	Psyllium husk	mixed herbs	25
Noureddin Soltanian [23]	2018	Iran	Clinical Nutrition ESPEN	patients with T2D, constipation	M/F	51	24	27	58	84	Psyllium cookie	Flaxseed and place cookie	20
Ayman S. Abutair [24]	2016	Palestine	Nutrition Journal	Type 2 diabetes patients	M/F	40	18	18		56	Psyllium supp	Diet without supp	10.5
James W. Anderson [25]	1988	USA	Arch Intern Med	M hypercholesterolemia.	M	28	14	14	47.6	70	Psyllium Hydrophilic Mucilloid	cellulose placebo	10.2
ARRIGO F.G. CICERO [26]	2007	Italy	Clinical and Experimental Hypertension	severe hyperlipoproteinemia; uncontrolled diabetes;	M/F	96	48	48	48	180	soluble psyllium husk powder	hydrolysed guar gum	7
Arrigo F.G. Cicero [27]	2010	Italy	Mediterr J Nutr Metab	Caucasian patients	M/F	96	48	48	48	180	soluble psyllium husk powder	hydrolysed guar gum	7
Fatemeh Pourbehi [28]	2020	Iran	International Journal of Women’s Health and Reproduction Sciences	non-diabetic women with PCOS.	F	58	24	24	24	56	psyllium	microcrystalline cellulose	10
Mark N. Feinglos	2013	USA	Bioactive carbohydrates dietary fibrel	patients with type-2 diabetes mellitus	M/F	23	15	8	61.8	140	psyllium	controlled by diet and/or an oral sulfonylurea,	3.4
Mark N. Feinglos [29]	2013	USA	Bioactive carbohydrates dietary fibrel	patients with type-2 diabetes mellitus	M/F	22	14	8	64.8	140	psyllium	controlled by diet and/or an oral sulfonylurea,	6.8
Mahdieh Kamalpour [15]	2017	Iran	Journal of Dietary Supplements	Patients with Type 2 Diabetes Mellitus		37	20	17	55.9	14	psyllium powder	LoCarb	7
Johnson W. McRorie [30]	2017	USA	Nutrition Today	subject	M/F	20	10	10	56.3	90	psyllium husk	wheat dextrin	10.2
Noureddin Soltaniana [31]	2018	Iran	Complementary Therapies in Medicine	patients with type 2 diabetes and chronic constipation	M/F	51	24	27	56.8	84	Psyllium cookie	Placebo cookie	20
Rosa Solàa [32]	2010	Spain	Elsevier	CVD	M/F	187	94	93	54.24	56	Po-husk	microcrystalline-cellulose	14
G Sartore [33]	2009	Italy	European Journal of Clinical Nutrition	type II diabetes.	M/F	40	20	20	61	56	psyllium	diet alone	10.5
Seyedeh Ferdows Jazayeri [34]	2021	Iran	HindawiEvidence-Based Complementary and Alternative Medicine	NAFLD	M/F	63	31	32	43.3	84	Plantago major	toasted flour powder	4
MISAEL URIBE [35]	1985	Mexico	Gastroenterology	Hepatic Encephalopathy and Diabetes Mellitus	M/F	8	4	4		14	psyllium Plantago		35 g/d fiber
Seyed Ali Ziai [14]	2005	Iran	Journal of Ethnopharmacology	diabetic patients		36	21	15	51.9	56	Psyllium	microcrystalline cellulose	10.2
Ricklefs Ka [36]	2017	USA	Obesity Medicine		M/F	17	8	9	58.5	56	ground psyllium	ground flaxseeds	9
Amjad Ali Bacha [37]	2022	Pakistan	Nutrition and Metabolic Insights	school teachers with central obesity	M/F	60	30	30	47	112	psyllium		10

N: number; In-N: intervention number; Pl-N; placebo number; IN: intervention; Pl: placebo; M: male; F: female

### Meta-analysis results

**Fig. 2:**
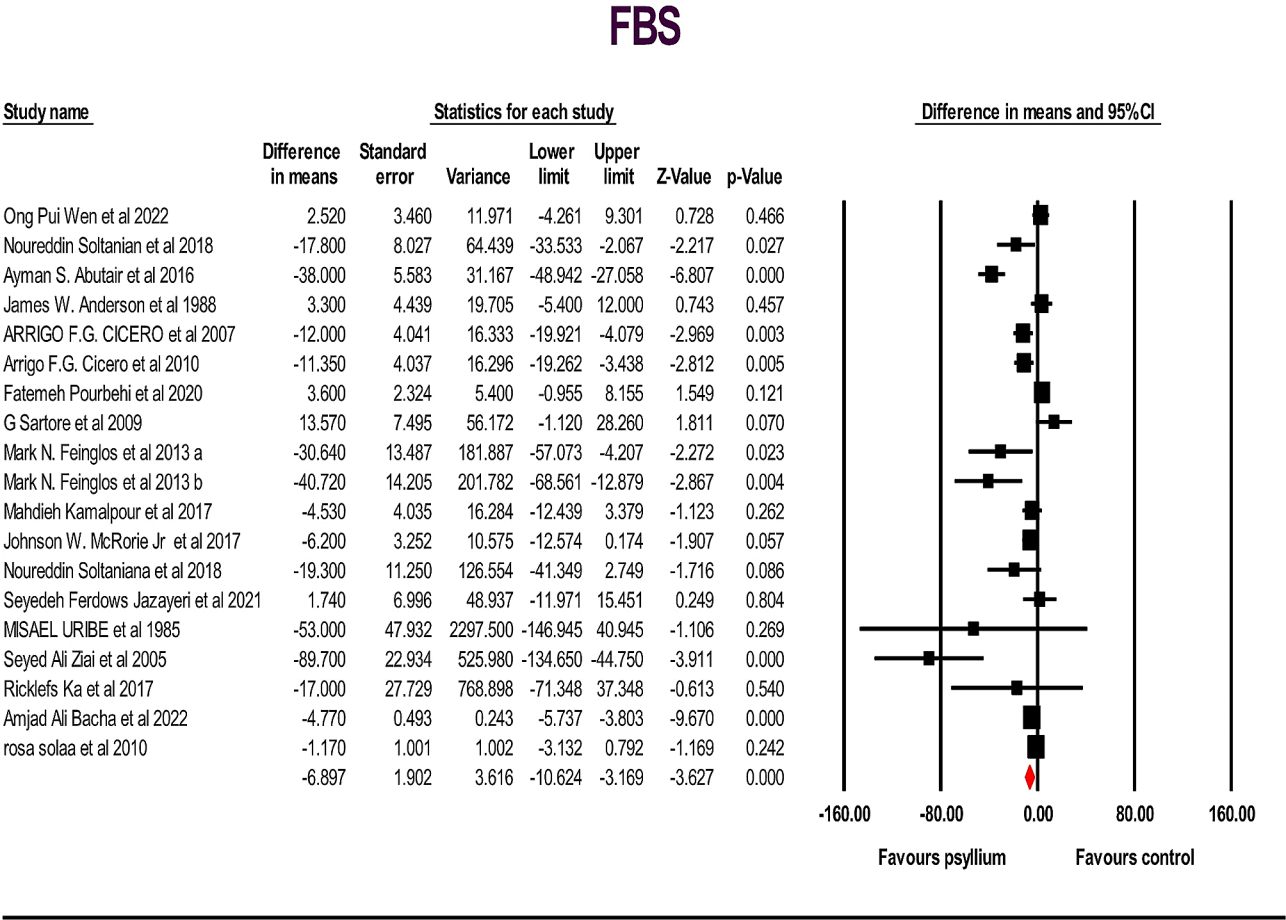
Forest plot illustrating weighted mean difference and 95% confidence intervals for the impact of psyllium consumption on fasting blood glucose

**Fig. 3:**
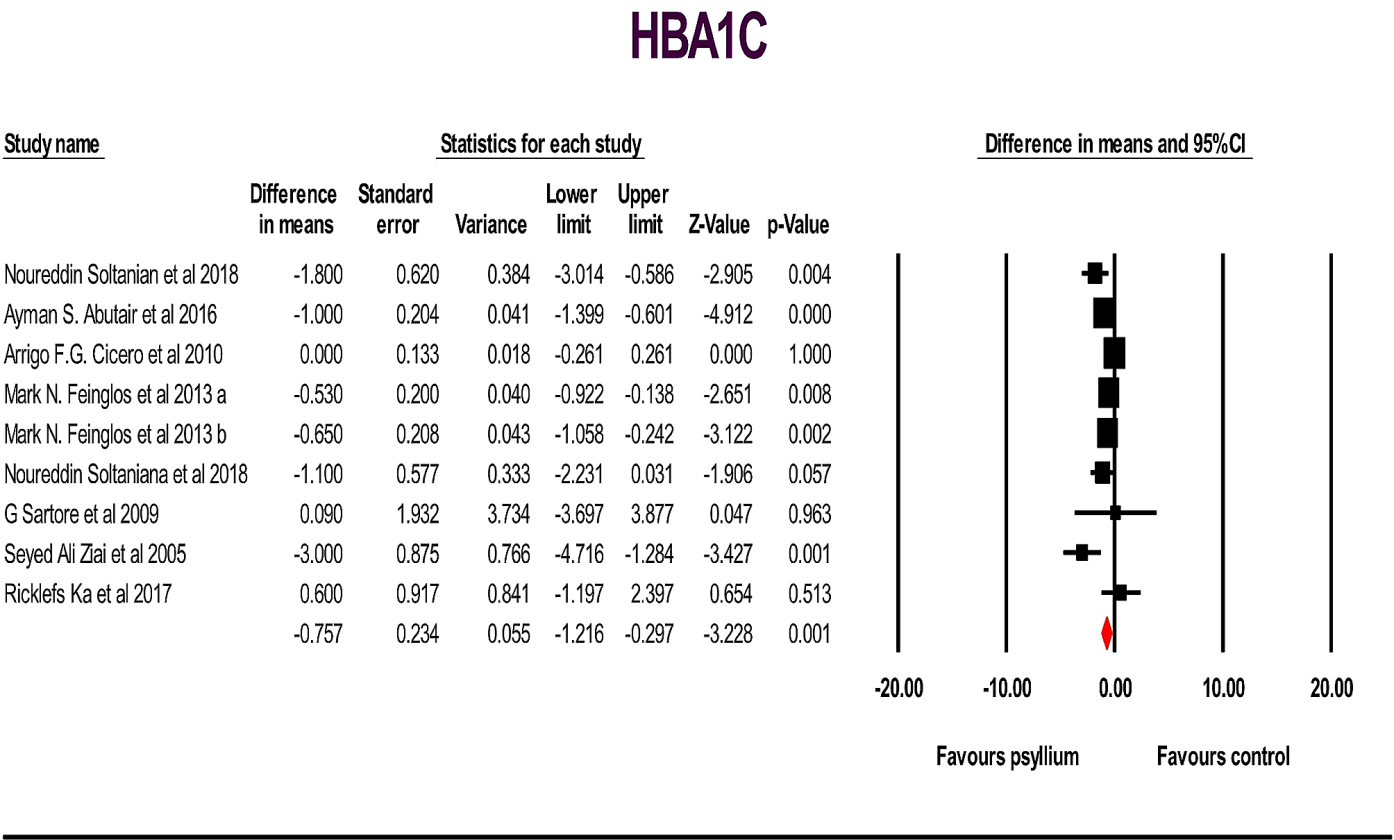
Forest plot illustrating weighted mean difference and 95% confidence intervals for the impact of psyllium consumption on HbA1c

**Fig. 4:**
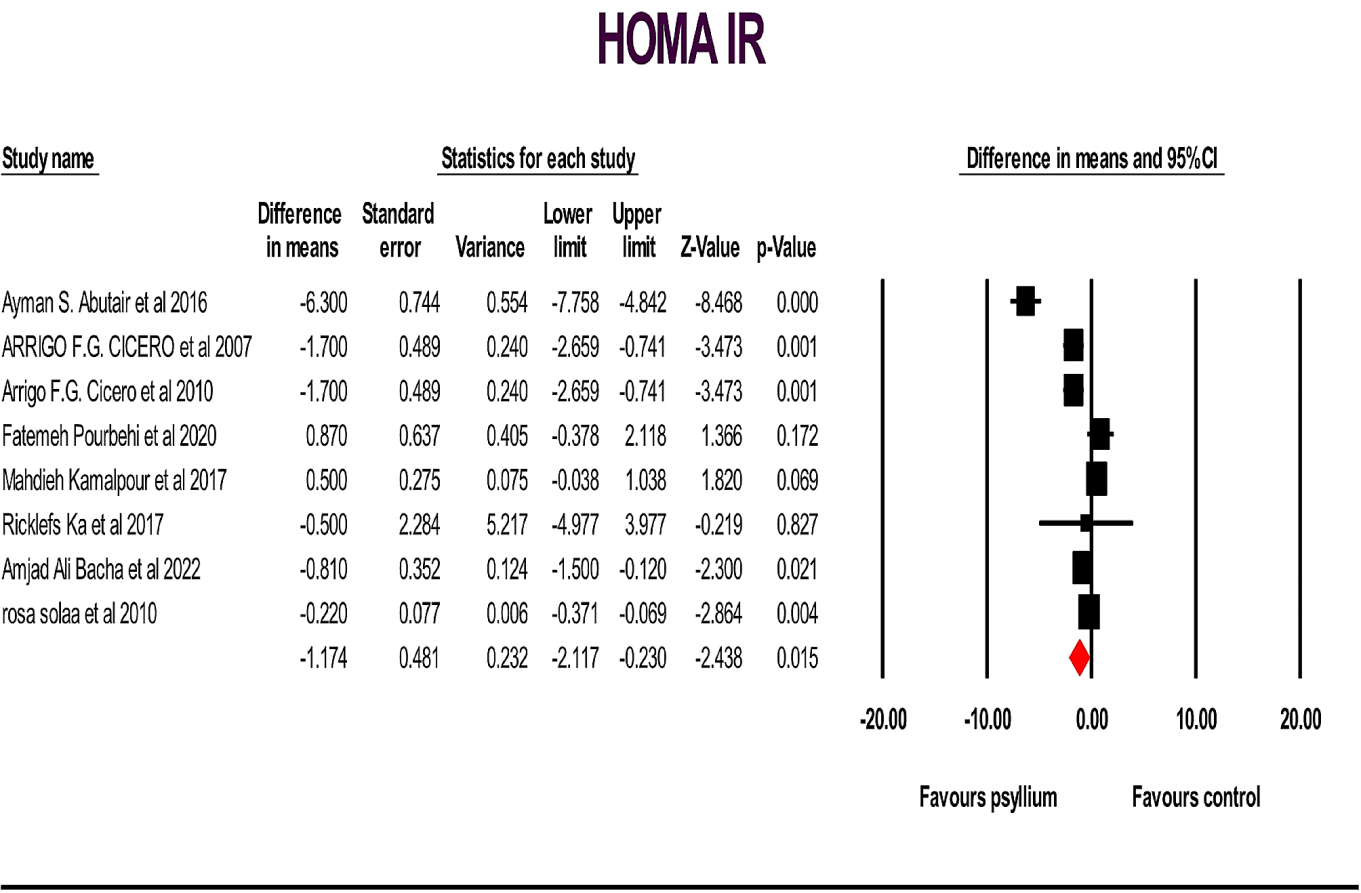
Forest plot illustrating weighted mean difference and 95% confidence intervals for the impact of psyllium consumption on HOMA IR

**Fig. 5:**
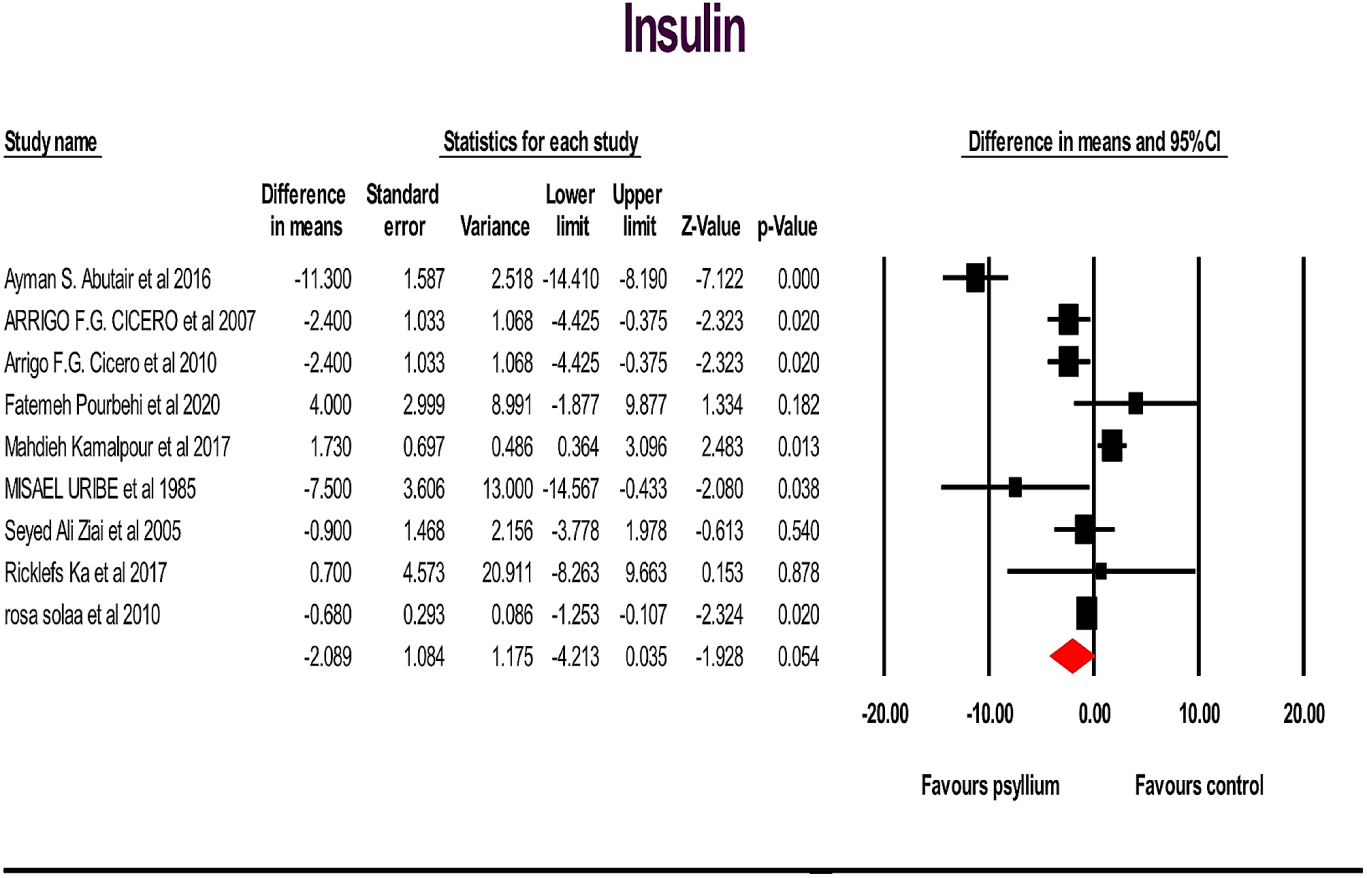
Forest plot illustrating weighted mean difference and 95% confidence intervals for the impact of psyllium consumption on insulin

A total of 19 studies, including 962 individuals (481 cases and 467 controls), examined the effects of psyllium supplementation on changes in FBS, HbA1c, HOMA IR and insulin levels. We used a random-effects model, which indicated a significant decrease in FBS, HbA1c, and HOMA IR levels, and a non-significant decrease in insulin levels, compared to the placebo FBS: (WMD): -6.89; 95% CI: -10.62, -3.16; *p* < .001) (Fig. 2), HbA1c: (WMD: -0.75; 95% CI: -1.21, -0.29; *p* < .001) (Fig. 3 ), HOMA IR: (WMD: -1.17; 95% CI: -2.11, -0.23; *p* < .05) (Fig. 4) and insulin: (WMD: -2.08; 95% CI: -4.21, -0.035; *p* > .05) ( Fig. 5). Just one study investigated QUIKI and because of this we didn’t check it. However, significant heterogeneity was noted for FBS: (I^2^ = 82.04%, *p* < .001); HbA1c: (I^2^ = 73.10%, *p* < .001), HOMA IR: (I^2^ = 87.27%, *p* < .001), and insulin: (I^2^ = 83.75%, *p* < .001). Sensitivity analyzes were performed using a one-study method (i.e., repeating the analysis excluding one study each time) to assess the influence of studies on the overall size effect [38, 39]. In the sensitivity analysis excluding a single study leads to changing the results. We perform sensitivity test on a study, we will exclude a study from the analysis, if *p*-value doesn’t change from significant (*p*-value < 0.05) to non-significant (*p*-value > 0.05) and vice versa, it means that the removal of that study does not affect the result of the study, and the result of the study is the same as the previous one, and if it changes, it means that remove Study will affect the result and the result depends on that study. The sensitivity analysis was robust for FBS: (WMD altered between − 4.49 and − 8.72), and HbA1c: (WMD altered between − 0.62 and − 0.89) because the results of the study did not change after removing each of the study. However, the result changes and becomes significant for the HOMA IR (WMD altered between − 1.49 and − 0.48) and insulin (WMD altered between − 2.75 and − 0.77), after removing two and three of the studies, respectively, in the event that the result was not significant before the removal of those studies (Supplementary Fig. 1).

### Risk of bias assessment, and GRADE assessment

Table 2^(14, 15, 17–32)^ displays the outcomes of the quality assessment of the trials. According to Cochrane Collaboration’s Upon scrutinizing the quality of all trials that were incorporated, 14 were appraised as having high quality, while the remaining four studies were evaluated as possessing medium quality, and one were appraised as having low quality. Table 3 contains the GRADE profile for the degree of certainty of the evidence. Due to serious limitations in imprecision and publication bias, FBS, HOMA IR, and HA1C were considered to be of moderate quality. Due to serious limitations in imprecision and publication bias, insulin was considered to be of low quality.

**Table 2:** Quality assessment According to Cochrane Collaboration’s Upon scrutinizing the quality of all trials

Article	Random sequence generation	Allocation concealment	Blinding participant and personal	Blinding of outcome assessment	Incomplete outcome data	Selective outcome reporting	Other potential threats to validity	General risk bias
Ong Pui Wen et al.	L	H	H	H	L	H	H	H
Noureddin Soltanian,	L	H	H	H	L	H	H	H
Ayman S. Abutair	L	H	H	H	L	L	H	H
James W Anderson,	H	H	L	H	L	H	L	H
ARRIGO F.G. CICERO	L	H	L	H	L	H	L	M
Arrigo F.G. Cicero	L	H	L	H	L	L	L	M
Fatemeh Pourbehi1	L	H	L	L	L	L	L	L
Mark N. Feinglos	H	H	L	L	L	H	H	H
Mark N. Feinglos	H	H	L	L	L	H	H	H
Mahdieh Kamalpour	H	H	H	H	L	H	L	H
Johnson W. McRorie Jr	L	H	H	H	L	H	H	H
Noureddin Soltaniana	L	H	H	H	H	H	H	H
Rosa Solàa	L	H	L	H	L	L	L	M
Seyedeh Ferdows Jazayeri	L	L	L	H	L	H	H	M
Amane Sheikh1	L	H	H	H	L	H	H	H
MISAEL URIBE	L	H	H	H	L	H	H	H
Seyed Ali Ziai	L	H	L	H	L	L	H	H
Ricklefs Ka	L	H	H	H	L	L	L	H
Amjad Ali Bacha	L	H	H	H	L	H	H	H

L: low

H: high

M: medium

**Table 3:** GRADE approach summary of findings and quality of evidence assessment

Outcome	No of studies	Design	Risk of bias	Inconsistency	Indirectness	Imprecision	Publication bias	Quality of evidence
FBS	19	**RCTs**	no serious ^a^	serious ^b^	Serious ^c^	no Serious ^d^	no serious ^e^	Moderate
insulin	9	**RCTs**	no serious	serious	Serious	serious	no Serious	Low
HOMA-IR	8	**RCTs**	no serious	serious	Serious	no Serious	no Serious	Moderate
HbA1c	9	**RCTs**	no serious	Serious	Serious	no Serious	no serious	Moderate

Using the GRADE system, the quality of the evidence is broken down into 4 categories (high, moderate, low, and very low). ^a^ the majority of the included studies were rated as having low risk of bias. ^b^ If the level of significant unexplained heterogeneity (I^2^ > 50%, *P* < .10, respectively) was present, the grade was downgraded. ^c^ If there were factors present that limited the generalizability of the results due to the participants, interventions, or outcomes, the grade would be downgraded. ^d^ the lower and upper bounds of the 95% confidence interval were < 0.95 and the optimal information size was not met, respectively. ^e^ If a funnel plot revealed evidence of publication bias, it was downgraded

### Subgroup analysis

We stratified studies based on baseline FBS, HbA1c, HOMA IR, and insulin levels (mean ± SD), psyllium dosage (g/d), study duration (days), and participants’ BMI. These analyses did not show any source of heterogeneity. Subgroup analyses illustrated diversities in the effects of psyllium on FBS: dosage subgroup with psyllium consumption less than vs. more than 10 g/d it showed significant difference for FBS (*p* value < 0.05). However, it was not significant when intervention duration was less than 50 days duration (*p* value > 0.05), HbA1c was not significant at dosages less than 10 g/d (*p* value > 0.05). HOMA IR and insulin were not significant at dosages less than and more than 10 g/d (*p* value > 0.05), respectively (Supplementary Figs. 2 and 3) .Because there was no differences in study duration about insulin, HbA1c, and HOMA IR, so dividing into subgroups was useless. The results of the subgroup analyses are summarized in (Table 4).

**Table 4:** Results of subgroup analyses for the effects of psyllium on FBS, HbA1c, HOMA IR, and insulin according to intervention

Variable		Number of comparisons	WMD (95% CI)	*P*-value	I squared	*p*- heterogeneity
**FBS**						
dose	< 10 g /day	7	-8.96(-13.67, -4.24)	< 0.001	69.93	< 0.001
	> 10 g/day	12	-3.97(-4.80, -3.15)	< 0.001	87.02	< 0.001
duration	< 50 day	3	-1.03(-8.26, 6.19)	0.77	32.35	0.22
	> 50 day	16	-8.01(-12.16, -3.87)	< 0.001	85.59	< 0.001
**Insulin**						
dose	< 10 g /day	4	-0.81(-3.57, 1.94)	0.56	82.11	< 0.001
	> 10 g/day	5	-3.26(-7.85, 1.33)	0.16	91.89	< 0.001
duration	< 50 day					
	> 50 day					
**HbA1c**						
dose	< 10 g /day	4	-0.32(-0.73, 0.09)	0.12	70.09	0.01
	> 10 g/day	5	-1.37(-2.03, -0.71)	< 0.001	38.74	0.16
duration	< 50 day					
	> 50 day					
**HOMA IR**						
dose	< 10 g /day	4	-0.88(-2.41, 0.63)	0.25	87.89	< 0.001
	> 10 g/day	4	-1.50(-3.25, 0.24)	0.09	95.80	< 0.001
duration	< 50 day					
	> 50 day					

FBS: fasting blood glucose; HbA1c: hemoglobin A1C; HOMA IR: **Homeostatic Model Assessment of Insulin Resistance**; WMD: weighted mean difference

### Publication bias

The assessment of publication bias is illustrated in plot (Supplementary Fig. 4). The Egger’s test indicated no evidence of publication bias in studies examining the effect of psyllium on FBS (*p* = .19). HbA1c (*p* = .19), HOMA IR (*p* = .24), and insulin (*p* = .40). The results of the publication bias analysis are shown in Table 5.

Begg’s test disclosed no evidence of publication bias in studies examining the effect of psyllium consumption on FBS (*p* = .10). HbA1c (*p* = .60), HOMA IR (*p* = .21), and insulin (*p* = .53) (Supplementary Fig. 4). Therefore, trim and fill analysis was performed, the FBS, HbA1c, HOMA IR, and insulin (no imputed study) were decreased after considering publication bias. The results of the publication bias analysis, as well as the overall effect, are shown in (Table 5).

**Table 5:** Results of publication bias for the effects of psyllium on FBS, HbA1c, HOMA IR, and insulin according to intervention

variable	Corrected effect size			Begg			Egger					Fail safe *n* test
	Study trimmed	WMD	CI 95%	KENDALL TAU	Z value	*P*-value 2 tailed	intercept	CI95%	T value	Df	*P*-value	*n*
FBS	0	-6.89	-10.62, -3.16	-0.26	1.60	0.10	-0.89	-2.30, 0.51	1.33	17.00	0.19	320.00
Insulin	0	-2.08	-4.21, 0.03	-0.17	0.62	0.53	-1.27	-4.67, 2.12	0.88	7.00	0.40	34.00
HbA1c	0	-0.75	-1.21, -0.29	-0.13	0.52	0.60	-1.61	-4.25, 1.02	1.44	7.00	0.19	78.00
HOMA IR	0	-1.17	-2.11, -0.23	-0.37	1.23	0.21	-2.15	-6.28, 1.96	1.27	6.00	0.24	73.00

FBS: fasting blood glucose; HbA1c: hemoglobin A1C; HOMA IR: **Homeostatic Model Assessment of Insulin Resistance**; CI: confidence interval; WMD: weighted mean difference

## Discussion

This systematic review and meta-analysis highlighted that a significant decrease in FBS, HbA1c, and HOMA IR levels was evident following psyllium consumption, vs. placebo. However, despite these findings, a significant amount of heterogeneity was indicated for FBS, HbA1c, HOMA IR, and insulin. The sensitivity analysis was robust for FBS and HbA1c because the results of the study did not change after removing each of the study. However, the result changes and becomes significant for the HOMA IR and insulin after removing two and three of the studies, respectively.

We categorized studies based on baseline FBS, HbA1c, HOMA IR, and insulin levels (mean ± SD), psyllium dosage (g/d), study duration (days), and participants’ BMI. Further subgroup analyses illustrated diversities in the effects of psyllium on FBS, HbA1c, HOMA IR, and insulin levels. For instance, in dosages less and more than 10 g/d, and intervention durations less than 50 days, were influential. For HbA1c, it was not significant in dosages less than 10 g/d. For HOMA IR and insulin, results were not significant in dosages less and more than 10 g/d, respectively. Psyllium dosage and duration of consumption had a remarkable linear effect on HbA1c was significant. Additionally, there was no publication bias evident in studies examining the effect of psyllium on FBS levels, HbA1c, HOMA IR, and insulin.

Previously, Xiao et al. reported that a significant reduction in FBS and HbA1c, which are indicators of glucose control, could be seen after supplementation with psyllium. Indeed, in the aforementioned study, the authors noted six studies, with 124 and 112 participants in the psyllium and control group, respectively, with overall results yielding a significant reduction in FBS levels and HbA1c [40] This study has been done on weight, body mass index, lipid profile, and glucose metabolism and it is a systematic review and meta-analysis of randomized controlled trials has like our study that been done only on diabetic people and it has examined just FBS and HbA1c. but our study has been done on glycemic indices and a systematic review and it has examined FBS, HbA1c, HOMA IR, and insulin. Indeed, similar findings were reported in Gibb et al. (2015), where the authors reported that postprandial blood glucose levels were significantly reduced [6]. Nevertheless, discrepant results have been reported across the literature; for instance, a randomized controlled trial on the impact of psyllium supplementation resulted in no significant effect on FBS vs. a carbohydrate reduction regimen [15]. However, with regards to Kamalpour et al., the lack of change reported may be attributable to the relatively short intervention period, i.e., two weeks [15]. Nevertheless, the authors did note a significant reduction in TNF-α and fasting plasma insulin, which have both been posited as mediators in numerous diabetes-associated complications [41, 42].

It has previously been suggested that consumption of psyllium before meals can significantly reduce fasting blood glucose levels and HbA1c levels [6]. Indeed, psyllium may be able to improve or manage glycemic control [40]. The mechanism of action for the reduction in blood sugar in patients with diabetes for psyllium is comparable to other soluble fibers. For instance, soluble fiber can result in a reduction in sugar absorption, which can, consequently, attenuate metabolic syndrome severity in diabetic patients. Psyllium may slow intestinal transit time and lead to an increased feeling of satiety, in addition to decreasing blood sugar and insulin requirements. The viscosity of soluble fiber is responsible for the slower absorption of macronutrients, and protection against digestive enzymes. Additionally, soluble fiber coats the intestinal surface, which prevents the passage of nutrients [24, 43–45]. Furthermore, consumption of foods with adequate fiber content elicits a lower insulin response and lower blood glucose levels. Indeed, psyllium can provoke changes in intestinal hormones and a subsequent reduction of glucose after meals [44].

### Strengths and limitations

This study has several strengths that should be acknowledged: (a) this was, to our knowledge, the first meta-analysis evaluating the effect of psyllium on fasting blood glucose, HbA1c, HOMA IR, and insulin; (b) we performed predefined subgroup analyses to identify sources of between-study heterogeneity; (c) we also performed a detailed sensitivity analysis; (d) To assess the degree of outcome evidence’s certainty, we used the GRADE method. However, against to the noted strengths, there are limitations that should be considered in the interpretation of our findings. For instance: (a) we obligatory limited the number of the included studies; (b) some of the included studies did not account the dietary intake, which is known to potentially affect blood glucose, HbA1c, HOMA IR, and insulin; (c) we had unidentified heterogeneity in several of the results; (d) the age range of included participants was wide. (e) The majority of the included studies were very small and used various psyllium types and doses during various intervention times; (f) different health status existed among the included subjects, and some significant confounders were left uncontrolled; (g) only 962 individuals—a relatively small number—are present in the literature used in this meta-analysis; (h) the most of the included studies had low quality.

## Conclusion

This systematic review and meta-analysis sought to investigate the influence of psyllium on HbA1c, fasting blood sugar (FBS), insulin, and Homeostatic Model Assessment of Insulin Resistance (HOMA IR), owing to the equivocal results in the extant literature. It seems that psyllium may improve glucose intolerance via reducing FBS, HbA1c, and HOMA IR levels. Therefore, we advise that psyllium be considered as a potential treatment option, if clinically appropriate.

### Electronic supplementary material

Below is the link to the electronic supplementary material.

Supplementary Material 1

Supplementary Material 2

## Data Availability

The datasets generated and/or analyzed during the current study are not publicly available due to some restrictions applied by ethics committee; but are available from the corresponding author on reasonable request.
