# Supplementary material for: The effect of psyllium on fasting blood sugar, HbA1c, HOMA IR, and insulin control: a GRADE-assessed systematic review and meta-analysis of randomized controlled trials
Source: BMC Endocr Disord. 2024 Jun 6;24:82. doi: 10.1186/s12902-024-01608-2 (PMC11155034; doi:10.1186/s12902-024-01608-2)
Supplement: Supplementary file 1 — Supplementary Material 1 [file 12902_2024_1608_MOESM1_ESM.docx]

### (a)

### (b)

### (c)

###

(d)

###

**Supplementary figure 1:** sensitivity analysis of psyllium on FBS (a), HbA1C (b), HOMA IR (c), and insulin (d)

(a)

### (b)

###

### (c)

### (d)

###

### Supplementary figure 2: dosage subgroup analyses for the effects of psyllium on FBS (a), HbA1C (b), HOMA IR (c), and insulin (d)

### Supplementary figure 3: duration subgroup analyses for the effects of psyllium on FBS

**(a)**

###

(b)

### (c)

###

(d)

###

**Supplementary figure 4:** Publication bias of psyllium on FBS (a), HbA1C (b), HOMA IR (c), and insulin (d)
